# Reversal treatment and clinical outcomes in acute intracranial haemorrhage associated with oral anticoagulant use: protocol of a planned systematic review and meta-analysis

**DOI:** 10.1136/bmjopen-2024-090357

**Published:** 2025-02-18

**Authors:** Mattias Tallroth, Linda Östlundh, András Büki, Yang Cao, Mia von Euler, Jakob O Ström

**Affiliations:** 1 School of Medical Sciences, Örebro University Faculty of Medicine and Health, Örebro, Sweden; 2 Örebro University Library, Örebro, Sweden; 3 Department of Neurosurgery, Örebro University Faculty of Medicine and Health, Örebro, Sweden; 4 Clinical Epidemiology and Biostatistics, School of Medical Sciences, Faculty of Medicine and Health, Örebro University, Örebro, Sweden; 5 Unit of Integrative Epidemiology, Institute of Enviromental Medicine, Karolinska Institute, Stockholm, Sweden; 6 Department of Neurology and Rehabilitation, Örebro University Faculty of Medicine and Health, Örebro, Sweden; 7 Department of General Medicine, Whangarei Hospital, Whangarei, New Zealand

**Keywords:** Intracerebral Haemorrhage, NEUROLOGY, NEUROSURGERY

## Abstract

**Abstract:**

**Introduction:**

Reversal treatment is commonly used for managing oral anticoagulant (OAC)-associated intracranial haemorrhages. Its effects on mortality are still understudied, particularly in various subtypes of intracranial haemorrhages. This systematic review and meta-analysis aims to synthesise the available data to study the impact of reversal therapies on mortality following various OAC-associated acute intracranial haemorrhages.

**Methods and analysis:**

This protocol follows the Preferred Reporting Items for Systematic reviews and Meta-Analyses (PRISMA) Protocols, and the final review will be reported in accordance with the PRISMA reporting guidelines. This systematic review and meta-analysis will include studies that assess contemporary reversal treatment in comparison to no reversal treatment, in cases of OAC-associated intracranial haemorrhage. Stratification will be performed for the types of bleeding as well as OAC at bleeding onset. Preliminary searches to determine search term inclusions were conducted in May–August 2024 in the electronic databases Embase, PubMed, Scopus and Web of Science without language and publication date restrictions. Randomised controlled studies, non-randomised controlled trials, and observational studies will be considered for the final meta-analysis. Three reviewers (MT, JOS and AB) will screen titles and abstracts, and one reviewer (MT) will subsequently conduct full-text screening.

Risks of bias will be assessed by MT using tools such as Risk of Bias 2, Risk Of Bias In Non-randomised Studies - of Interventions and the Newcastle-Ottawa Scale. Heterogeneity among the study results will be assessed using the I² statistic. If appropriate, a random-effects meta-analysis model will be performed. Subgroup analyses and meta-regression (if applicable) will be performed to assess sources of heterogeneity among (1) intracranial haemorrhage types, (2) OAC drugs and (3) study types, with randomised controlled trials being the primary focus.

**Ethics and dissemination:**

Ethical approval is not needed as this project involves previously published data. We intend to publish the results in a peer-reviewed journal.

**PROSPERO registration number:**

CRD42024556420.

**STRENGTHS AND LIMITATIONS OF THIS STUDY**

- A broad scope aimed at synthesising data stratified for specific, understudied subgroups of interest.
- A robust search strategy, designed with a librarian, with the objective of achieving a comprehensive and highly sensitive literature overview.
- Authors with significant clinical expertise in the topic.
- There may be a lack of randomised controlled trials for some subgroups.
- In the case of observational studies, there may be difficulties in sufficiently addressing the effects of confounding by indication.

## Introduction

### Rationale

 Use of vitamin K antagonists (VKAs) or direct oral anticoagulants (DOACs) is a significant risk factor for intracranial haemorrhage and poor outcomes once bleeding occurs.^1^,^4^ A primary driving factor is purported to be early haematoma expansion exacerbated by an attenuated secondary haemostasis.^5^ Reversal agents aimed at restoring haemostasis, thus logically appear as promising treatments.^6^,^8^ The gold standard of VKA reversal is currently a combination of prothrombin complex concentrate (PCC) and vitamin K.^9^ Multiple studies have shown excellent and quick reversal, yet these findings have not been consistently translated into improved clinical outcomes in intracerebral haemorrhage.^10 11^ PCC is also used for DOAC reversal and in particular in activated factor 10 (FXa) inhibitor-associated bleeds.^8^ Evidence for improved clinical outcomes is weak with markedly inconsistent findings.^12^,^14^ Similarly, andexanet alfa, a novel FXa inhibitor decoy drug, despite pharmacological superiority to PCC has failed to achieve convincing clinical success in terms of improved outcomes.^15^,^17^ Finally, although positive results have been reported with idarucizumab, a dabigatran-specific antidote,^6^ there is a lack of large-scale studies with outcome data.^18 19^

The American Stroke Association recommends VKA reversal with PCC as a Class I recommendation in acute intracerebral haemorrhage management, primarily relating to its robust association with the prevention of haematoma expansion.^1120^,^22^ Nonetheless, effects on mortality or functional outcomes are less validated.^10 11^ Both andexanet alfa and idarucizumab currently fall under Class IIa recommendations, primarily due to questions regarding their efficacy.^15 16 20^ DOAC treatment is rapidly increasing, which has resulted in a shift towards more DOAC-associated intracerebral haemorrhages, further highlighting the need for more DOAC-specific reversal studies.^23 24^

Although intracerebral haemorrhages constitute the majority of all intracranial bleeds,^13^ reversal treatment could theoretically be an option for any oral anticoagulant (OAC)-associated intracranial haemorrhage. It is, however, far more uncertain whether haemostatic treatment poses significant benefits in the treatment of all types of intracranial haemorrhage.^25^ A meta-analysis from 2022 assessing DOAC reversal in relation to any intracranial haemorrhage showed overwhelmingly positive results in terms of pharmacological reversal.^12^ Whether this translated to improved patient outcomes was nevertheless unclear, and the study was not designed to distinguish between specific intracranial haemorrhage types. Similar data are lacking regarding traumatic intracranial bleeds and subarachnoid haemorrhages.

### Objectives

**Figure 1:**
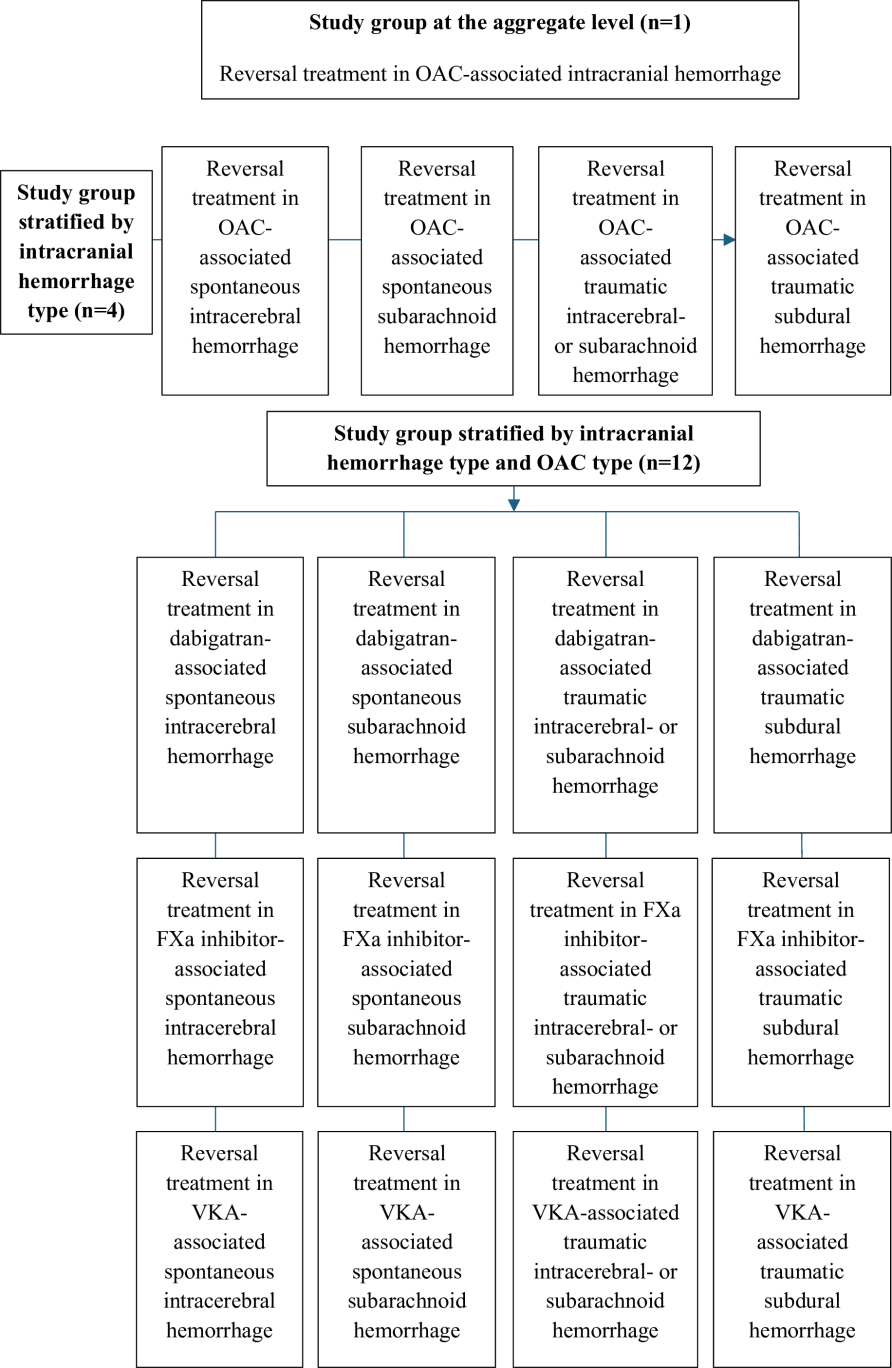
Flowchart illustrating the scope of the project including the study group at the aggregate level extending down to the individual strata. FXa, activated factor 10; OAC, oral anticoagulant; VKA, vitamin K antagonist.

This protocol is in accordance with the Preferred Reporting Items for Systematic reviews and Meta-Analyses (PRISMA) Protocols extension and the final review will be reported according to the PRISMA reporting guidelines.^26 27^ The primary aim of this work is to systematically review the existing literature and to conduct a meta-analysis on studies investigating the impact of reversal treatment on mortality in cases of different subtypes of intracranial haemorrhages associated with OAC use. This will be performed at the aggregate level, that is, reversal treatment for any included intracranial haemorrhage and OAC, but also in each subgroup of interest. Thus, the included bleeding types (spontaneous intracerebral haemorrhage, spontaneous subarachnoid haemorrhage, traumatic intracerebral haemorrhage or subarachnoid haemorrhage and traumatic subdural haemorrhage) will be studied separately as well as in relation to each OAC reversal group (reversal of dabigatran, FXa inhibitors and VKAs, respectively) (figure 1). This approach will be used as intracranial haemorrhage types are heterogeneous in their nature, and the reversal of different classes of OACs might be associated with different outcomes. The intervention of reversal treatment will be compared with non-reversal (conservative) treatment, where the latter could be argued to not reflect contemporary clinical practice. However, it would be argued that there is merit in such comparisons considering the potential risk of thromboembolic complications associated with these treatments, thus creating a need to delineate whether the effects on mortality are net positive.

## Methods

### Eligibility criteria

This protocol has been registered to PROSPERO (registration number: CRD42024556420). The primary aim is to meta-analyse randomised controlled trials. However, non-randomised controlled trials and observational studies will also be included, as these represent a consecutive sample of patients. Other study types, such as case reports, will be excluded to limit selection bias. Studies must include a comparison of patients administered reversal treatment versus a control group not receiving reversal treatment. Disease severity and age must be controlled in the statistical analysis. Studies of patients≥16 years of age diagnosed with acute OAC-associated intracranial haemorrhage will be included. Intracranial haemorrhage could be defined either explicitly, that is, brain scans, if reported in text, or will be assumed to be implicitly defined by brain scans if the diagnostic method is not specified in the article. Intracranial haemorrhages included are spontaneous intracerebral haemorrhage, spontaneous subarachnoid haemorrhage, traumatic intracerebral haemorrhage or subarachnoid haemorrhage or traumatic subdural haemorrhage. Spontaneous intracranial haemorrhages may include all aetiologies but traumatic. Intracranial haemorrhages are considered acute if not explicitly stated otherwise. Patients must have reported the use of either dabigatran, an FXa inhibitor or a VKA at intracranial haemorrhage diagnosis. Studies where the reporting of OAC exposure is not explicitly stated, alternative to, for example, laboratory testing, thereby acknowledging the potential for unknown compliance will not be excluded, as it is believed this reflects clinical practice. If the source used to determine the drug use is not stated, it is assumed that data were retrieved from medical records primarily, nonetheless, displaying intentions to treat. Only contemporary reversal treatments will be included and are limited to PCC (3-factor or 4-factor), vitamin K, idarucizumab and andexanet alfa. To be included, there needs to be a study arm with participants intended to not receive reversal treatment (the control). However, in cases of small (<20%) contaminations of either study arm, that is, patients with inappropriate drug exposure, the record will be included and the effects will be explored in sensitivity analyses. The outcome measure mortality must be included.

Studies involving individuals below the age of 16 years will be excluded. Studies involving primarily epidural bleeds will be excluded as they commonly affect a patient group rarely treated with OAC. Similarly, studies involving primarily non-traumatic subdural bleeds will be excluded due to the heterogeneous nature of non-traumatic subdural bleeds, resulting in substantial variations in management. Mixed group studies of intracranial haemorrhages, which contain a subpopulation of either epidural bleeds or non-traumatic subdural bleeds, will however be included. Studies primarily including any reversal treatment not listed above, that is, inferior, non-conventional or outdated treatments, will be excluded partly or altogether.

### Information sources

A systematic search for literature will be performed from inception in four electronic databases (Embase, PubMed, Scopus and Web of Science) without publication year, demographical, type of setting or language limitations. An artificial intelligence-based translation tool will be used for the title and abstract screening process. In cases of non-English language studies included in the subsequent full text review, the assistance of an appropriate translator will be sought. Preliminary searches in electronic databases to determine and evaluate search term inclusions and search syntax for the review were conducted in May–August 2024. Sources for grey or unpublished materials will not be covered in the search.

### Search strategy

The preliminary search string was systematically developed with the help of PubMed and PubMed’s Medical Subject Headings (MeSHs). It will later consistently be repeated in all databases. Entry terms for all MeSHs were assessed for inclusion. All search terms will be searched in a combination of the fields ‘Title’, ‘Abstract’ and in the MeSH/Thesaurus (when available) and truncated as needed for the best possible results. Results from presearches are illustrated in the online supplemental appendix.

In addition to the database search, the reference lists of the finally included studies will be screened. The complete search will be updated ahead of the final manuscript preparation. A search log with all search-technical details, search term inclusion, results, dates, notes for all searches and the search update will be appended to the review to allow easy reproduction and appraisal of the search strategy.

### Study records

#### Data management

All records found in the database search will be exported to the systematic review software Covidence (Covidence systematic review software, Veritas Health Innovation, Melbourne, Australia), where automatic de-duplication will be performed, and the software is set up for blinded screening. Cabell’s Predatory Report will be consulted to ensure the academic status of the finally selected papers published in open access journals.^28^ The results from the screening and selection process will be recorded in a PRISMA flowchart.

#### Selection process

Three reviewers (MT, JOS and AB) will screen titles and abstracts based on the preset inclusion and exclusion criteria. A fourth reviewer (MvE) will be consulted to resolve disagreements regarding the inclusion of an abstract. One reviewer (MT) will independently apply the eligibility criteria to conduct full-text screenings of all subsequently included records. All records meeting the inclusion criteria will have their reference lists assessed anterogradely using Scopus by MT to ensure literature saturation.

#### Data collection process and data items

One reviewer (MT) will independently extract the following data from all records satisfying the eligibility criteria: first author’s name, study design, methodology, participant demographics, baseline participant characteristics (age, sex, OAC indication and disease severity), intracranial haemorrhage type, OAC type, reversal therapy administration including drug administered, follow-up status and mortality (dichotomous, yes/no). Estimates and 95% CIs for the outcome (mortality) will be obtained either directly from the data, independently calculated or, in cases of missing information, requested from the authors. A modified version of The Cochrane Collaboration’s data collection form will be used for data extraction. The data extraction forms have been piloted prior to the final searches.

### Outcome

The primary outcome will be mortality with a maximum follow-up period of 90 days. The rationale for including mortality up to 90 days is to capture the potential effects of thromboembolic complications on mortality. The associations between the primary outcome and the treatments in the included studies should be evaluated using measures such as relative risk/risk ratio (RR), OR, HR or incidence rate ratio (IRR). If OR was used, it will be converted to RR, provided that the required information is available. The authors intend to perform separate analyses for randomised controlled trials and observational studies.

### Quality assessment of individual studies

The quality of individual studies will be assessed by MT using tools such as Risk of Bias 2 for randomised controlled trials, Risk Of Bias In Non-randomised Studies - of Interventions for non-randomised controlled trials and the Newcastle-Ottawa scale for observational studies.

### Data analysis

The data analysis for this systematic review and meta-analysis will involve several steps to synthesise the findings from included studies, assess the impact of reversal treatment on mortality in cases of intracranial haemorrhage associated with OAC use and explore potential heterogeneity among studies.

#### Meta-analysis methodology

RR, HR or IRR along with their SEs, 95% CI or other measures representing their variance will be collected or calculated for each study. HR will be considered equivalent to RR for the purpose of pooling in a meta-analysis.

Heterogeneity among the study results will be assessed using the I² statistic, where I² values of 25%, 50% and 75% are considered low, moderate and high heterogeneity, respectively.

In cases of substantial heterogeneity (I²> 50%), a random-effects model that accounts for the heterogeneity among studies will be applied. This model assumes that the observed effects vary across studies due to differences in study populations, interventions and outcomes. The effects from individual studies, as well as the overall effects, will be visualised using forest plots.

#### Handling missing values

For studies missing the SE of RR, HR or IRR, SEs from 95% CI will be derived to ensure precise estimation of effect sizes and variances. If both SE and 95% CI are unavailable, imputing SEs using estimates from studies involving similar populations or attempting to obtain the missing data directly from the study authors will be considered, thereby improving the precision of this analysis.

#### Publication bias

To assess the presence of publication bias, funnel plots and Egger’s test will be used initially. As per the guidelines of Cochrane, Egger’s test is recommended to be used when 10 or more studies are included. In cases where studies are fewer than 10, the authors will rely on visual inspection, that is, funnel plots.^29^ Funnel plots visually inspect the distribution of effect sizes against a measure of study precision, revealing asymmetries that may indicate publication bias. Egger’s test provides a statistical measure of funnel plot asymmetry.

In cases where publication bias is detected, the Trim-and-Fill method will be employed as a corrective measure. This method assumes that the funnel plot of a meta-analysis should be symmetrical in the absence of publication bias. It ‘trims’ (removes) asymmetric studies to make the plot symmetrical and then ‘fills’ by adding imputed studies that mirror the removed studies on the opposite side of the effect size axis. This adjusted analysis estimates the number of missing studies and provides a revised pooled effect estimate that accounts for the presumed missing studies due to publication bias.

#### Subgroup analyses

Subgroup analyses and meta-regression (if applicable) will be performed to explore potential sources of heterogeneity, such as differences in intracranial haemorrhage types, OAC drugs involved and study design.

Subsequent analyses will stratify the included studies by the type of intracranial haemorrhage: spontaneous intracerebral haemorrhage, spontaneous subarachnoid haemorrhage, traumatic intracerebral haemorrhage or subarachnoid haemorrhage, and traumatic subdural haemorrhage.

Further stratification will be performed based on the type of OAC involved: dabigatran, FXa inhibitors and VKAs. If data are sufficient, subgroup analyses based on participants’ age will be conducted.

To inform the interpretation of the stratified results and the reliability of the overall synthesis, the I² statistic will be used to quantify heterogeneity within each stratified analysis.

#### Sensitivity analysis

A sensitivity analysis will incorporate the leave-one-out approach, which systematically excludes one study at a time from the meta-analysis and recalculates the pooled effect estimate with the remaining studies. This process is iterated so that each study is excluded once. Another sensitivity analysis will be performed by excluding studies with a high risk of bias and those that significantly contribute to heterogeneity. This will help assess the robustness of the overall findings and determine the influence of individual studies and potential sources of bias. Finally, a sensitivity analysis will be performed by excluding studies with small (<20%) contaminations in either study arm due to inappropriate reversal treatment exposures.

#### Reporting of results

Tables will be used to summarise the study characteristics, outcomes and findings of the risk of bias assessment.

The results of the meta-analysis will be presented in forest plots to visually display the effect sizes and 95% CIs from individual studies, as well as the pooled effect size.

The findings from subgroup analyses, sensitivity analyses and assessments of publication bias will also be reported in detail to provide a comprehensive understanding of the impact of reversal treatment on mortality in OAC-associated intracranial haemorrhage.

The results will be interpreted in the context of the quality and heterogeneity of the evidence, the biological plausibility of the findings and the potential impact on clinical practice and future research.

#### Software and tools

Data analysis will be performed using statistical software R V.4.33 (R Foundation for Statistical Computing, Vienna, Austria) and Stata V.18.0 (StataCorp, College Station, Texas, USA).

### Meta-bias(es)

Meta-biases include: publication bias, where positive results are more likely published; selective reporting bias, involving the preferential reporting of significant outcomes; language bias, potentially excluding non-English studies; citation bias, where studies with significant findings are more readily identified; time-lag bias, affecting the speed of publication based on the nature of results; multiple publications bias, where data from a single study published across various articles might be redundantly included. To mitigate these biases, the methodology encompasses comprehensive literature searches across multiple databases and languages, predefined study inclusion criteria, diligent checks for data duplication and statistical assessments (see the ‘Publication bias’ section). These measures ensure the reliability and applicability of our meta-analytical conclusions.

### Confidence in cumulative evidence

The confidence in cumulative evidence of this systematic review and meta-analysis will be assessed according to the Grading of Recommendations Assessment, Development and Evaluation system. This will involve evaluating several critical dimensions including the risk of bias, consistency of results across studies, directness of evidence to the research question, precision of effect estimates and the potential for publication bias.^30^ These evaluations provide a structured approach to grade the quality of evidence as high, moderate, low or very low, reflecting the degree of confidence in the synthesised conclusions. This assessment aids in forming robust recommendations for clinical practice and policy-making, underscoring the importance of high-quality, relevant and precise evidence in informing healthcare decisions.

## Discussion

This protocol outlines a planned systematic review and meta-analysis on the effects of contemporary reversal treatment on clinical outcomes in intracranial haemorrhage. Unlike previous meta-analyses, the authors aim to synthesise data for various bleeding types as well as for multiple OAC types. The authors believe that such an overview is needed, as reversal treatment for oral anticoagulation has been considered *standard care*. OAC use is a growing clinical concern as these drugs’ therapeutic indications are continuously expanding, now encompassing far beyond ischaemic stroke prevention.^31^ Current Swedish register data suggest that one in four intracerebral haemorrhages, the most prevalent form of intracranial haemorrhage, occur during OAC treatment.^13 23^ PCC was in a recent propensity-score weighted observational study not associated with improved outcome versus conservative treatment in DOAC-associated intracerebral haemorrhage.^32^ The effects of idarucizumab, the dabigatran-specific antidote, have been shown, but its effects on mortality have yet to be demonstrated.^6 12^ Furthermore, the recently published ANNEXA-I study showed no effect on clinical outcome as the positive effect of haemostatic control of andexanet alfa in FXa inhibitor-associated intracerebral haemorrhage was balanced with a higher risk of thromboembolic complications.^17^ Ultimately, the high cost of these drugs further underscores the need to justify their use.

### Ethics and dissemination

Ethical approval is not needed as this project involves previously published data. The authors intend to publish the results in a peer-reviewed journal.

## supplementary material

10.1136/bmjopen-2024-090357 online supplemental file 1
