# Supplementary material for: Reversal treatment and clinical outcomes in acute intracranial haemorrhage associated with oral anticoagulant use: protocol of a planned systematic review and meta-analysis
Source: BMJ Open. 2025 Feb 18;15(2):e090357. doi: 10.1136/bmjopen-2024-090357 (PMC11836858; doi:10.1136/bmjopen-2024-090357)
Supplement: online supplemental file 1 [file bmjopen-15-2-s001.docx]

**Source:** PubMed.

**Search date:** 2024-05-23.

**Search technical specifications:** all search terms are searched in combination of the search fields: title, abstract and MeSH (when available). No filters or limitations were applied.

| Search string | **Results and notes** |
| --- | --- |
| ("Intracranial Hemorrhages"[Mesh:NoExp] OR "Cerebral Hemorrhage"[Mesh] OR "Intracranial Hemorrhage, Hypertensive"[Mesh] OR "Intracranial Hemorrhage, Traumatic"[Mesh:NoExp] OR "Brain Hemorrhage, Traumatic"[Mesh] OR "Hematoma, Subdural"[Mesh:NoExp] OR "Hematoma, Subdural, Acute"[Mesh] OR "Hematoma, Subdural, Intracranial"[Mesh] OR "Subarachnoid Hemorrhage, Traumatic"[Mesh] OR "Subarachnoid Hemorrhage"[Mesh] OR "Hemorrhagic Stroke"[Mesh] OR "Hemorrhages, Intracranial"[Title/Abstract] OR "Intracranial Hemorrhag*"[Title/Abstract] OR "Intracranial Haemorrhag*"[Title/Abstract] OR "Hemorrhage, Intracranial"[Title/Abstract] OR "Haemorrhage, Intracranial"[Title/Abstract] OR "Brain Hemorrhag*"[Title/Abstract] OR "Brain Haemorrhag*"[Title/Abstract] OR "Intracerebral Hemorrhag*"[Title/Abstract] OR "Intracerebral Haemorrhag*"[Title/Abstract] OR "Hemorrhage, Cerebral"[Title/Abstract] OR "Haemorrhage, Cerebral"[Title/Abstract] OR "Cerebral Hemorrhag*"[Title/Abstract] OR "Cerebral Haemorrhag*"[Title/Abstract] OR "Intracranial Hematoma*"[Title/Abstract] OR "Intracranial Haematoma*"[Title/Abstract] OR "Subdural Hematoma*"[Title/Abstract] OR "Subdural Haematoma*"[Title/Abstract] OR "Hemorrhage, Subdural"[Title/Abstract] OR "Haemorrhage, Subdural"[Title/Abstract] OR "Hemorrhages, Subdural"[Title/Abstract] OR "Subdural Hemorrhag*"[Title/Abstract] OR "Subdural Haemorrhag*"[Title/Abstract] OR "Hematoma, Intracranial Subdural"[Title/Abstract] OR "Hemorrhage, Subarachnoid"[Title/Abstract] OR "Haemorrhage, Subarachnoid"[Title/Abstract] OR "Subarachnoid Hemorrhag*"[Title/Abstract] OR "Subarachnoid Haemorrhag*"[Title/Abstract] OR "Hemorrhagic Strok*"[Title/Abstract] OR "Haemorrhagic Strok*"[Title/Abstract] OR "Stroke, Hemorrhagic"[Title/Abstract] OR "Stroke, Haemorrhagic"[Title/Abstract] OR "Brain bleed*"[Title/Abstract] OR "Intracranial bleed*"[Title/Abstract] OR "Intracerebral bleed*"[Title/Abstract] OR "Cerebral bleed*"[Title/Abstract] OR "Subdural bleed*"[Title/Abstract] OR "Subarachnoid bleed*"[Title/Abstract] OR "Brain hematoma*"[Title/Abstract] OR "Intracerebral hematoma*"[Title/Abstract] OR "Cerebral hematoma*"[Title/Abstract] OR "Subarachnoid hematoma*"[Title/Abstract] OR "Brain haematoma*"[Title/Abstract] OR "Intracerebral haematoma*"[Title/Abstract] OR "Cerebral haematoma*"[Title/Abstract] OR "Subarachnoid haematoma*"[Title/Abstract]) AND ("Hemostatics"[Mesh:NoExp] OR "Anticoagulant Reversal Agents"[Mesh] OR "Anticoagulation Reversal"[Mesh] OR "Vitamin K"[Mesh:NoExp] OR "Vitamin K 1"[Mesh] OR "Hemostatic*"[Title/Abstract] OR "Haemostatic*"[Title/Abstract] OR "Antihemorrhagic*"[Title/Abstract] OR "Antihaemorrhagic*"[Title/Abstract] OR "PPSB"[Title/Abstract] OR "Proplex"[Title/Abstract] OR "Octaplex"[Title/Abstract] OR "Praxbind"[Title/Abstract] OR "BI-655075"[Title/Abstract] OR "BI 655075"[Title/Abstract] OR "ADABI-FAB"[Title/Abstract] OR "andexanet"[Title/Abstract] OR "r-antidote"[Title/Abstract] OR "Phytonadione"[Title/Abstract] OR "Vitamin K*"[Title/Abstract] OR "Phytomenadione"[Title/Abstract] OR "Konakion"[Title/Abstract] OR "Prothrombin complex*"[Title/Abstract] OR "Idarucizumab"[Title/Abstract] OR "PRT064445"[Title/Abstract] OR "Antidote*"[Title/Abstract] OR "Reversal*"[Title/Abstract] OR "Ondexxya"[Title/Abstract] OR "PCC"[Title/Abstract] OR "Confidex"[Title/Abstract] OR "Praxbind"[Title/Abstract]) | Results:  3,203  The search was conducted without language or year restrictions |
