# Supplementary material for: Reversal treatment and clinical outcomes in acute intracranial haemorrhage associated with oral anticoagulant use: protocol of a planned systematic review and meta-analysis
Source: BMJ Open. 2025 Feb 18;15(2):e090357. doi: 10.1136/bmjopen-2024-090357 (PMC11836858; doi:10.1136/bmjopen-2024-090357)
Supplement: Supplementary file 2 [file bmjopen-2024-090357.reviewer_comments.pdf]

PEER REVIEW HISTORY

BMJ Open publishes all reviews undertaken for accepted manuscripts. Reviewers are asked to complete a checklist review form and are provided with free text boxes to elaborate on their assessment. These free text comments are reproduced below.

ARTICLE DETAILS

Title (Provisional)

Reversal Treatment and Clinical Outcomes in Acute Intracranial Hemorrhage Associated with Oral Anticoagulant use - Protocol of a Planned Systematic Review and Meta-analysis

Authors

Tallroth, Mattias; Östlundh, Linda; Büki, András; Cao, Yang; von Euler, Mia; Strom, Jakob O

VERSION 1 - REVIEW

**Reviewer**	**1**
**Name**	**Ghannam, Malik**
**Affiliation**	**University of Iowa Health Care**
**Date**	**23-Sep-2024**
**COI**	**None**

Title: would add "study level meta-analysis"

Methods:

1. " Patients must

have reported the use of either dabigatran, an FXa inhibitor, or a VKA at intracranial

hemorrhage diagnosis" would define the last time the patient received the drug? within 48 hours? also did you choose INR limit? did you choose factor Xa level as a biomarker?

2. did you exclude aneurysmal SAH? vascular malformation? please clarify

3. define the time period for the search (from which month/year to which month/year)?

4. was this registered in prospero? if so please include

5. you stated you included retrospective data, did you have specific number of patients that need to be met in the study to be included? please clarify

6. did you use Liberian to help in the search startegy? would clarify in the methods

7. "If OR was used, it will be converted to RR provided that the required information is available." to be more consistent, may be better to analyze retrospective study using OR separately from RCTs using RR.

8. would define the author/s who did the ROB assessment in the methods.

9. Egger's test, you need to have 10 or more studied for each tested outcome, make sure this is clarified in your methods.

10. I would suggest to do subgroup analysis if feasible based on age

Results: the results and forest plots are lacking from this article, please address.

**Reviewer**	**2**
**Name**	**Chaudhary, Rahul**
**Affiliation**	**University of Pittsburgh**
**Date**	**27-Sep-2024**
**COI**	**None**

The proposed study addresses a significant gap in the literature regarding the management of oral anticoagulant-associated intracranial hemorrhage. Its comprehensive scope, including various intracranial hemorrhage types and oral anticoagulant classes, enhances its potential impact on clinical practice. However, the novelty could be further enhanced by including additional outcome measures beyond mortality like hematoma expansion.

Specific comments:

In Methods,

- The handling of non-English language studies and studies with mixed populations needs clarification.

- The primary outcome being limited to mortality may miss other important clinical outcomes.

- Clarify the approach to studies with mixed populations.

VERSION 1 - AUTHOR RESPONSE

Reviewer: 1

Comment from reviewer:

Title: would add "study level meta-analysis"

Thank you for this suggestion which would add further clarity to the title. However, the present title adheres to the PRISMA-P guidelines and is quite long in its current state. We

would thus prefer to not add “study level” as most meta-analyses are study level and it could thus be argued to be implicitly stated.

Methods:

1. " Patients must have reported the use of either dabigatran, an FXa inhibitor, or a VKA at intracranial hemorrhage diagnosis" would define the last time the patient received the drug? within 48 hours? also did you choose INR limit? did you choose factor Xa level as a biomarker?

Reply:

Thank you for the comment. We agree that having such data would be desirable. However, according to the results from preliminary searches, it does not appear feasible to require specific information attesting to the degree of oral anticoagulant drug use. We suspect that imposing restrictions such as only including studies where *e.g.* timing of DOAC intake is explicitly stated, would greatly limit the number of available studies. Ultimately, as stated on lines 236-240, one could consider our “lenient” inclusion criteria an “intention to treat”. We believe this reflects clinical practice, where definite drug exposure in the acute setting may remain unknown, and where treatment decisions are based on medical records.

2. did you exclude aneurysmal SAH? vascular malformation? please clarify

Reply:

Thank you for noting this which needed further clarification. We intend to include all etiologies of SAH under the term “nontraumatic SAH”. This has been clarified by the statement “Spontaneous intracranial hemorrhages may include all etiologies but traumatic”. This also applies to nontraumatic intracerebral hemorrhage. See lines 233-234.

3. define the time period for the search (from which month/year to which month/year)?

Reply:

Thank you for this remark which further improves transparency. All searches were performed from the inception of the respective database, which has been further clarified in line 259.

We’ve also specified that the these, preliminary searches, were conducted starting in May and ending in August 2024 (lines 62-63 and 265).

4. was this registered in prospero? if so please include

Reply:
Yes, we included this statement in the title page “PROSPERO registration number: CRD42024556420” (line 46). The registration was performed before the submission of the protocol. This has been further clarified in line 221.

5. you stated you included retrospective data, did you have specific number of patients that need to be met in the study to be included? please clarify

Reply:
Thank you for this comment which highlights a concept that might need further clarification. No, we did not conduct any a priori power analysis. We suspect that there will be insufficient data to answer some of the prespecified PICOs (e.g. reversal treatment in some specific subgroups of bleeds) and the review will thus serve as a scoping review in those cases. Furthermore, our aim is to study a consecutive sample of patients with intracranial hemorrhage diagnosis during a given study period (starting from the inception of the respective databases). Resulting from this, we aim to exclude sutudies with few participants, *e*.g. case reports, in order to mitigate selection bias. This has been further clarified, see lines 223-225.

6. did you use Liberian to help in the search startegy? would clarify in the methods

Reply:
Yes, Linda Östlundh is an experienced librarian. This is mentioned in the affiliations section as well as briefly in the Article Summary section (lines 13 and 101).

7. "If OR was used, it will be converted to RR provided that the required information is available." to be more consistent, may be better to analyze retrospective study using OR separately from RCTs using RR.

Reply:

Thank you for this remark and we agree that your suggestion is cleaner. We have clarified that we intend to perform separate analyses for randomised controlled trials and observational studies, thus not mixing OR and RR (see lines 317-318).

8. would define the author/s who did the ROB assessment in the methods.

Reply:

Thank you for making this observation. The ROB assessment will be performed by Mattias Tallroth which has been clarified (see lines 69 and 321).

9. Egger's test, you need to have 10 or more studied for each tested outcome, make sure this is clarified in your methods.

Reply:

Thank you for highlighting this. We have added a segment clarifying when Egger's test will be performed as well as how we will handle a scenario with less than 10 included studies (lines 349-351).

10. I would suggest to do subgroup analysis if feasible based on age

Reply:

Thank you for this comment. According to preliminary searches, it is uncertain whether there will be sufficient data available for subgroup analyses based on age. However, this will be performed if possible and we've added a statement regarding this (lines 369-370).

Results: the results and forest plots are lacking from this article, please address.

Reply:

As this manuscript details a protocol for a planned systematic review and meta analysis, the results and corresponding forest plots are yet to be produced. We've added a sentence clarifying that these will be presented in the final manuscript. See lines 338-340.

Reviewer: 2
Comments to the Author:
The proposed study addresses a significant gap in the literature regarding the management of oral anticoagulant-associated intracranial hemorrhage. Its comprehensive scope, including various intracranial hemorrhage types and oral anticoagulant classes, enhances its potential impact on clinical practice. However, the novelty could be further enhanced by including additional outcome measures beyond mortality like hematoma expansion.

Specific comments:
In Methods,
- The handling of non-English language studies and studies with mixed populations needs clarification.

Reply:
Thank you for this remark. Although the manuscript states there will be "no language limitations", we did not detail the handling of non-English language studies. We will use an artificial intelligence-based translation tool for the title and abstract screening process. In cases of non-English language studies included for subsequent full text review, we will consult an appropriate translator. This has been clarified in lines 261-265.

Our initial plan was to exclude studies of nontraumatic subdural bleeds as well as epidural bleeds. As preliminary searches indicate that these bleeding types may, in some studies, constitute a small percentage of a larger group of intracranial hemorrhage, we plan to include said bleeding types when grouped under the umbrella term of intracranial hemorrhages. As these bleeds are generally associated with good outcomes, relatively to other intracranial hemmorhage subtypes, this should not significantly alter the direction of our results. Studies involving only nontraumatic subdural bleeds or epidural bleeds will be excluded all together. This has been clarified in the paragraph consisting of lines 248-254.

- The primary outcome being limited to mortality may miss other important clinical outcomes.

Reply:
Thank you for the comment as well as the suggestion of including hematoma expansion as an additional outcome measure. We agree that there are many potential outcomes of interest. We did consider to include a safety outcome, mainly thromboembolic complications, but also other outcomes such as hematoma expansion or functional outcome (modified Rankin Scale or Barthel Index). However, in light of the already large scope of the present study including >10 PICOs (Figure 1), the inclusion of another outcome would effectively double the amount of PICOs. Our concern is that that this would possibly render the study to being too scattered.

Mortality is a distinct outcome measure which we proposed would be generally presented when comparing groups. We have choosen mortality as the sole outcome as *i)* this has to our knowledge not been the subject of any previous meta-analyses and as *ii)* some studies have failed to link reversal treaments' effects on preventing hematoma expansion to improved mortality (on a group level). This was shown in *Connolly Stuart J., Sharma Mukul, Cohen Alexander T., et al. Andexanet for Factor Xa Inhibitor–Associated Acute Intracerebral Hemorrhage. New England Journal of Medicine. 2024 May 15;390(19):1745–55*), where it can be suspected that thromboembolic complciations counterbalance the beneficial effects of preventing hematoma expansion, ultimately nullifying any effects on mortality. A similar concept has been shown with fresh frosen plasma, which is, however, not the subject of the present study, where effects on preventing hematoma expansion did not translate to reduced mortality *Mayer SA, Brun NC, Begtrup K, Broderick J, Davis S, Diringer MN, et al. Efficacy and Safety of Recombinant Activated Factor VII for Acute Intracerebral Hemorrhage. New England Journal of Medicine. 2008 May 15;358(20):2127–37*.

Ultimately, we believe that there is an argument for focusing on mortality specifically. To be able to conduct appropriate subgroup analyses with stratifications for bleeding types as well as oral anticoagulant types, we plan to limit the scope to this outcome only. This rationale was briefly discussed in the Discussion section (lines 451-454).
- Clarify the approach to studies with mixed populations.

Reply:
Thank you for this remark. Our plan of adressing studies including various etiologies of intracranial hemorrhage has been clarified on the previous page in this document. However,

there is also the matter of potential contaminations among treatment arms, *i.e.* patients with inappropriate reversal treatment, in *i)* the intervention group and *ii)* in the control group.

Preliminary searches suggest that some studies include *e.g.* an intervention group consisting of a small portion of fresh frosen plasma recepients along with a larger group of prothrombin complex recepients. We plan to tolerate such groups given that no more than 20% receive an alternative intervention (such as fresh frosen plasma). The rationale is that the categorical exclusion of said studies would greatly reduce any potential records available for the meta-analysis. As it has been shown that prothrombin complex concentrate is pharmacologically superior to fresh frosen plasma, the introduction of a small group of fresh frosen plasma users could possibly attentuate a positive effect of prothrombin complex concentrate. The effects of including such studies will be explored in sensitivity analyses.

Preliminary searches also suggest that some studies will include a control group consisting of *e.g.* a portion of patients receiving fresh frosen plasma. Such studies will also be included, under the same conditions as stated above, and the effects will similarly be explored with sensitivity analyses. In some cases, it appears that fresh frosen plasma will contaminate both the intervention and control group thus making it possible that its confouding effects are counterbalanced.

Finally, as we impose relatively strict inclusion criteria including that the studies' statistical analyses must account for differences in baseline age and bleeding severeity, we believe that such contaminations as described above should be tolerated in order to not severely limit the sensitivty of the search. We aim to discuss said impurities pertaining to the treatment arms, in the discussion.

We've added a section detailing this, see lines 242-245 and lines 380-382.
